# Population genetic testing and *SERPINA1* sequencing identifies unidentified alpha-1 antitrypsin deficiency alleles and gene-environment interaction with hepatitis C infection

**DOI:** 10.1371/journal.pone.0286469

**Published:** 2023-08-31

**Authors:** Bryce A. Schuler, Lisa Bastarache, Janey Wang, Jing He, Sara L. Van Driest, Joshua C. Denny

**Affiliations:** 1 Department of Pediatrics, Vanderbilt University Medical Center, Nashville, Tennessee, United States of America; 2 Department of Biomedical Informatics, Vanderbilt University Medical Center, Nashville, Tennessee, United States of America; 3 Department of Biostatistics, Vanderbilt University Medical Center, Nashville, Tennessee, United States of America; 4 Department of Medicine, Vanderbilt University Medical Center, Nashville, Tennessee, United States of America; 5 *All of Us* Research Program, National Institutes of Health, Bethesda, Maryland, United States of America; 6 National Human Genome Research Institute, National Institutes of Health, Bethesda, Maryland, United States of America; Medizinische Fakultat der RWTH Aachen, GERMANY

## Abstract

Alpha-1 antitrypsin deficiency (AATD), a relatively common autosomal recessive genetic disorder, is underdiagnosed in symptomatic individuals. We sought to compare the risk of liver transplantation associated with hepatitis C infection with AATD heterozygotes and homozygotes and determine if *SERPINA1* sequencing would identify undiagnosed AATD. We performed a retrospective cohort study in a deidentified Electronic Health Record (EHR)-linked DNA biobank with 72,027 individuals genotyped for the M, Z, and S alleles in *SERPINA1*. We investigated liver transplantation frequency by genotype group and compared with hepatitis C infection. We performed *SERPINA1* sequencing in carriers of pathogenic AATD alleles who underwent liver transplantation. Liver transplantation was associated with the Z allele (ZZ: odds ratio [OR] = 1.31, p<2e^-16^; MZ: OR = 1.02, p = 1.2e^-13^) and with hepatitis C (OR = 1.20, p<2e^-16^). For liver transplantation, there was a significant interaction between genotype and hepatitis C (ZZ: interaction OR = 1.23, p = 4.7e^-4^; MZ: interaction OR = 1.11, p = 6.9e^-13^). Sequencing uncovered a second, rare, pathogenic *SERPINA1* variant in six of 133 individuals with liver transplants and without hepatitis C. Liver transplantation was more common in individuals with AATD risk alleles (including heterozygotes), and AATD and hepatitis C demonstrated evidence of a gene-environment interaction in relation to liver transplantation. The current AATD screening strategy may miss diagnoses whereas *SERPINA1* sequencing may increase diagnostic yield for AATD, stratify risk for liver disease, and inform clinical management for individuals with AATD risk alleles and liver disease risk factors.

## Introduction

Alpha-1 antitrypsin deficiency (AATD) is an autosomal recessive Mendelian genetic disorder caused by reduced abundance or dysfunction of the alpha-1 antitrypsin (A1A) protein, encoded by *SERPINA1*. Decreased functional A1A exposes host tissues to non-specific neutrophil proteases leading to tissue damage; this loss of function is the primary mechanism of lung disease in AATD [1, 2]. Accumulation of abnormally folded A1A has also been shown to contribute to cellular damage in hepatocytes and lead to AATD-associated liver dysfunction [2, 3]. Tissue damage in AATD is most prominent in the lungs and liver; recognized features include emphysema, chronic obstructive pulmonary disease (COPD), cirrhosis, increased risk for hepatocellular carcinoma, neonatal liver dysfunction, and in rare cases, vasculitis and panniculitis [2].

There are a variety of pathogenic alleles in the *SERPINA1* gene. Screening for AATD consists of quantifying A1A in the blood and, if the level of A1A is low, gel-electrophoresis-based protease inhibitor (PI) typing determines which *SERPINA1* alleles are present. The reference allele in the *SERPINA1* gene is referred to as “M”; the most common pathogenic alleles are “Z” (more severe) and “S” (more common but milder AATD) [4]. Within each PI type are a variety of alleles that confer different protein abundance and/or function that confers risk to lung and/or liver damage, respectively, with ZZ homozygotes typically having the most severe AATD and other risk allele combinations having milder AATD. Additionally, there are null alleles whose mRNA transcripts undergo nonsense mediated decay and thus are not detected on PI typing but should be detected as a low A1A level [4].

Estimations of AATD prevalence in European ancestry populations are between 1:2000–7000 [5–8] while the carrier frequency of the Z allele in some populations is as high as 1:25 [8]. Despite this relatively high prevalence, some estimate that fewer than two percent of people with symptomatic AATD have a diagnosis [9]. Additionally, the delay between symptom onset and diagnosis is seven to ten years by some measures [9]. Diagnosis is complicated by incomplete penetrance and variable expressivity, especially with age [5, 10]. Screening is recommended for individuals with COPD regardless of age or ethnicity; a family history of COPD, liver disease, or AATD; chronic liver disease of unknown etiology; or severe, treatment refractory asthma [8, 9, 11]. Despite these recommendations for broad screening, AATD underdiagnosis and delayed diagnosis are persistent problems [12].

Beyond the marginal benefit provided by enzyme replacement therapy, providing an AATD diagnosis can alter the disease course [11, 12]. There are known environmental exposures that can exacerbate or accelerate tissue damage secondary to AATD in both heterozygotes and homozygotes including smoking, environmental pollution, respiratory infections, obesity, non-steroidal anti-inflammatory drugs, and alcohol [13–17]. There are additional environmental factors, like viral hepatitis, whose impact on exacerbating AATD-related tissue damage is debated [13, 18–24]. Early AATD diagnosis can lead to an avoidance of these environmental exposures and an attenuation of symptom manifestation [12, 25]. Research is in progress to determine if early detection can improve overall morbidity and mortality associated with AATD [12], but detection and diagnosis of this genetic disease can also lead to cascade screening of family members, pre-symptomatic diagnosis, and risk factor modification such as decreased smoking [11, 12, 25].

We sought to identify undiagnosed cases of AATD among individuals who had undergone liver transplantation. We compared the frequency of liver transplantation between AATD risk alleles and a non-genetic cause of liver failure, hepatitis C infection. Additionally, we hypothesized that the current screening strategy that consists of A1A levels with reflex PI typing will miss rare cases of AATD. Here, we confirmed that individuals homozygous and heterozygous for AATD risk alleles both have increased rates of liver transplantation, demonstrated that there is a gene-environment interaction between AATD-risk alleles and hepatitis C infection in the context of need for liver transplantation, and identified individuals who are compound heterozygous for rare pathogenic variants in *SERPINA1* whose symptoms overlap with AATD but lack a formal diagnosis, at times despite having been clinically tested.

## Materials and methods

### Study design

This is a retrospective cohort study utilizing an Electronic Health Record (EHR)-linked DNA biobank. Deidentified EHR data were collected over a 31-year period at our tertiary care center institutional biobank and included 72,027 European ancestry individuals genotyped for the M, Z, and S alleles in *SERPINA1*. We hypothesized that frequency of liver transplantation would correlate with AATD-related genetic risk and sought to determine how that risk compared to the risk of transplantation with hepatitis C infection. We hypothesized that AATD would be over-represented in individuals who had undergone liver transplantation and that those individuals could provide the opportunity to identify undiagnosed AATD through *SERPINA1* sequencing.

### Electronic health record data

This study was reviewed and the need for ethical approval was waived by the Vanderbilt Institutional Review Board (IRB) and deemed as non-human subject research. Individuals with DNA specimens and linked, de-identified electronic health record (EHR) data were obtained from BioVU, Vanderbilt’s DNA biobank [26]. As this is non-human subject research on de-identified EHR data, informed consent was deemed not necessary by the Vanderbilt IRB to access these data. All work contained in this manuscript was performed in accordance with the Declaration of Helsinki and in accordance with relevant guidelines and regulations. Data collected included International Classification of Diseases (ICD) codes for diagnoses, laboratory values, age, sex, and duration of EHR data available. ICD9 and ICD10 codes were aggregated into their respective phenotype codes or “phecodes” [27–29] to determine the presence or absence of phenotypes that could be represented by multiple ICD codes. Presence of a single phecode was sufficient to count as having the phenotype, as previously described [30, 31]. Similarly, a clinical diagnosis of AATD was defined as having at least one ICD code for AATD (phecode 270.34). In addition to demographic data, we determined who had undergone liver transplantation (phecode 573.2), had a diagnostic code for hepatitis C infection (phecode 070.3), and had an A1A level.

### *SERPINA1* genotype data

Genotype data were obtained from DNA specimens in BioVU [26, 32]. Included individuals had previously undergone genome-wide genotyping using the Illumina Infinium^®^ Expanded Multi-Ethnic Genotyping Array plus custom content (VUMC BioVU MEGAEX). After Illumina’s GenomeStudio Genotyping Module, rigorous QC measures were conducted in which data were filtered by both sample and SNP call rates > = 95% and minor allele frequency >1%; gender mismatch was reviewed and discrepancies resolved, if possible, and data removed if unresolvable; concordances of genotype with HapMap and duplicated samples and concordance of allele frequency with gnomAD database were checked. Admixture 1.3 software [33] was used to calculate ancestry fraction (Q). Samples with Q> = 0.8 were selected as European ancestry. 72,027 individuals of European ancestry had both EHR data in the Synthetic Derivative and genotype data available for study from BioVU. Genotypes in *SERPINA1* were collected and included the reference “M” allele and the most common pathogenic alleles, “Z” and “S.”

### Gene-environment interaction

We identified individuals who had undergone liver transplantation and those who had evidence of hepatitis C infection in their EHR. Hepatitis C was chosen as a relatively common environmental exposure that increases risk for liver failure requiring transplantation. The presence or absence of an ICD code for hepatitis C infection was more readily ascertainable than exposures like tobacco or alcohol which change in quantity and frequency over time and are inconsistently reported in EHR data.

### Whole exome sequencing and *SERPINA1* variant analysis

MS or MZ heterozygotes genotype groups who underwent liver transplantation were chosen for whole exome sequencing (WES) to determine if there were other pathogenic alleles that were not captured on the genotyping array; 140 of those individuals had sufficient quantity and quality of DNA for sequencing. WES reads were processed via Illumina dynamic read analysis for genomics (DRAGEN), a GATK-based germline short variant discovery pipeline in Illumina BaseSpace Sequencing Hub. DRAGEN’s “hard filter” and SNPs call rate >90% were applied to remove low quality variants. WES analysis focused on variants in *SERPINA1* that were rarer than the most common, known pathogenic allele (i.e., the S allele). Literature review was used to determine if the rare sequence variants identified were known to be previously reported pathogenic variants in AATD.

### Chart review

Chart review was performed on the six compound heterozygotes for rare variants in *SERPINA1* and seven individuals who underwent liver transplantation, were heterozygous for a variant in *SERPINA1*, and had evidence of hepatitis C infection. Chart review investigated the sex, age, BMI, alcohol- and tobacco-use history, COPD status, and A1A level measurements and PI typing, if available, and included full access to de-identified content of the EHRs.

### Statistical analyses

Data analyses were conducted in R Version 3.6.1 (Boston, Massachusetts, USA). Means of continuous variables were compared with a Wilcoxon rank sum test. Confidence intervals for percentages were calculated using the “BinomCI” function and the Wilson continuity correction. P-values were calculated with Fisher’s exact test (two-sided, 95% confidence interval) to compare discrete count data. They were not corrected for multiple comparisons. Linear and logistic regressions were performed with the “glm” function and were corrected for age, sex, and number of years of EHR data available.

## Results

### Demographics of study cohort

There were 72,027 individuals of European ancestry for whom both EHR and genotype data were available (Table 1). 56% were female, and 94 individuals had a clinical diagnosis of AATD (0.1%, or one in 766). The mean age at the time of data analysis in those with a diagnosis was 49.4 years compared to 52.1 years in those without an AATD diagnosis (p = 0.24). These trends were consistent when the cohort was divided into genotype groups: 46–57% of each genotype group was female and there was no significant difference in age between those with and without an AATD diagnosis (Table 1). AATD diagnoses were more prevalent in the groups with at least one copy of the Z allele: MZ with nine clinical diagnoses among 2,704 individuals (0.3%), SZ with 19 out of 41 (13.5%), and ZZ with 64 out of 85 (75.3%) (Table 1).

**Table 1:** Genotype, sex, age at time of analysis, and AATD diagnosis status of study individuals. Data for the entire study population are in the first column of the table (“All”). Individuals are categorized by genotype in the subsequent columns. Standard deviation (SD) is indicated in parentheses next to the mean values. P-values were calculated with a Wilcoxon rank sum test; there were no significant differences between mean age with and without a diagnosis.

	Genotype Group						
	All	MM	MS	MZ	SS	SZ	ZZ
Number (% of Total)	72,027 (100%)	62,598 (87%)	6,331 (8.8%)	2,704 (3.8%)	168 (0.23%)	141 (0.20%)	85 (0.12%)
Female (% of Genotype)	40,329 (56%)	35, 076 (56%)	3,578 (57%)	1,469 (54%)	85 (51%)	82 (58%)	39 (46%)
Mean Age of All Individuals (SD)	52.1 (22.0)	52.1 (22.0)	52.3 (21.8)	53.1 (21.9)	54.5 (20.9)	51.0 (23.1)	47.5 (23.2)
Number with AATD Diagnosis (% of Genotype)	94 (0.1%)	1 (0.002%)	1 (0.02%)	9 (0.3%)	0 (0%)	19 (13.5%)	64 (75.3%)
Mean Age, with Diagnosis (SD)	49.4 (21.4)	—	—	61.1 (12.8)	—	51.5 (17.0)	47.1 (23.3)
Mean Age, No Diagnosis (SD)	52.1 (22.0)	52.1 (22.0)	52.3 (21.8)	51.3 (21.9)	54.5 (20.9)	50.9 (23.9)	48.9 (23.3)

### Liver transplantation associated with AATD risk genotypes and hepatitis C infection

**Fig 1:**
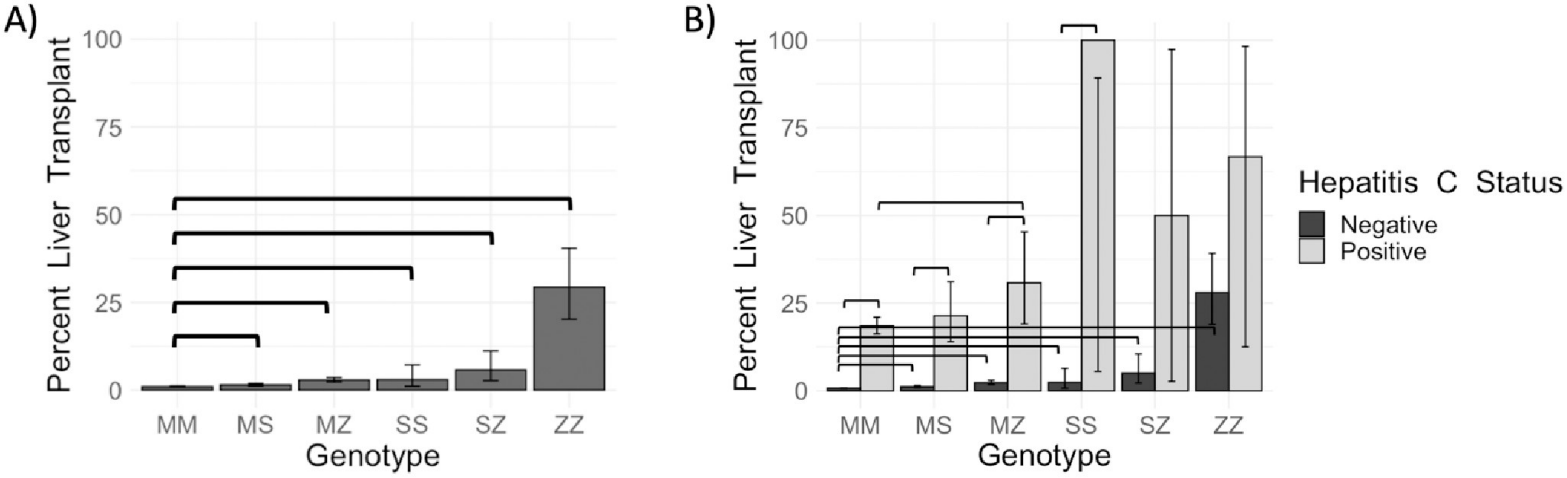
Percent of individuals undergoing liver transplantation increases with AATD risk genotypes (A), hepatitis C infection, and the combination of those risks (B). Hepatitis C infection and liver transplantation status were determined by the presence or absence of the respective phecode in the EHR. Confidence intervals were calculated using the continuity corrected Wilson interval and p-values were calculated using a Fisher’s exact test. Significant differences (p < 0.05) are indicated by brackets.

We assessed the rates of liver transplantation across *SERPINA1* genotype groups (Fig 1A). When compared to the reference genotype group (“MM”), a greater proportion of those with at least one pathogenic AATD allele had undergone liver transplantation (Fig 1A).

Comparison of the observed risk of liver transplantation across AATD-risk genotypes and hepatitis C infection status revealed the latter was associated with a significantly higher percent of liver transplantation in the absence of any AATD-risk alleles (18.5% in MM hepatitis C positive vs. 0.8% in MM hepatitis C negative, p<2.20e^-16^) (Fig 1B and Table 2). With both an AATD-risk allele and hepatitis C infection, the percentage of individuals who underwent liver transplant increased in all groups. This increase was significant in the MS (p = <2.20e^-16^), MZ (p = 2.16e^-13^), and SS (p = 2.98e^-02^) genotype groups but not significant in the SZ (p = 0.11) and ZZ (p = 0.21) groups (Fig 1B and Table 2). There was a significant association between liver transplantation and each non-reference genotype when compared to the reference MM genotype (Table 3). The association between hepatitis C infection and liver transplantation was significant (OR = 1.20, p< 2.00e^-16^). The interaction between non-reference *SERPINA1* genotypes and hepatitis C for the outcome of liver transplantation was significant for all groups (MS: OR = 1.03, p = 0.018, MZ: OR = 1.11, p = 6.92e^-13^, SS: OR = 2.21, p = 6.05e10^-15^, SZ: OR = 1.31, p = 1.69e10^-4^, and ZZ: OR = 1.23, p = 4.70e10^-4^; Fig 1B, Tables 2 and 3).

**Table 2:** Values corresponding to Fig 1B showing rates of liver transplantation by genotype and hepatitis C infection status. Hepatitis C infection and liver transplantation status were determined by the presence of the respective phecode in the EHR. Confidence intervals (CI) were calculated using the continuity corrected Wilson interval and p-values were calculated using a Fisher’s exact test. Comparisons for the outcome of liver transplantation were made between hepatitis C positive and hepatitis C negative individuals within a genotype group (Hepatitis C Comparison) and between genotypes within a hepatitis C infection status group (Genotype Comparison).

Hepatitis C Status	Genotype	Liver Transplant	Total	Percent (CI)	Hepatitis C Comparison	P-Value	Genotype Comparison	P-Value
Positive	MM	190	1,029	18.5% (16.2–21.0%)	MM (Hep C+) vs. MM (Hep C-)	<2.20E-16	—	—
Negative	MM	492	61,569	0.8% (0.7–0.9%)	—	—	—	—
Positive	MS	21	98	21.4% (14.0–31.1%)	MS (Hep C+) vs. MS (Hep C-)	<2.20E-16	MS (Hep C+) vs. MM (Hep C +)	4.99E-01
Negative	MS	75	6,233	1.2% (1.0–1.5%)	—	—	MS (Hep C-) vs. MM (Hep C -)	1.62E-03
Positive	MZ	16	52	30.8% (19.1–45.3%)	MZ (Hep C+) vs. MZ (Hep C-)	2.16E-13	MZ (Hep C+) vs. MZ (Hep C +)	4.41E-02
Negative	MZ	62	2,652	2.3% (1.8–3.0%)	—	—	MZ (Hep C-) vs. MZ (Hep C -)	1.90E-12
Positive	SS	1	1	100% (5.5–89.2%)	SS (Hep C+) vs. SS (Hep C-)	2.98E-02	SS (Hep C+) vs. SS (Hep C +)	1.85E-01
Negative	SS	4	167	2.4% (0.8–6.4%)	—	—	SS (Hep C-) vs. SS (Hep C -)	4.64E-02
Positive	SZ	1	2	50.0% (26.7–97.3%)	SZ (Hep C+) vs. SZ (Hep C-)	1.11E-01	SZ (Hep C+) vs. SZ (Hep C +)	3.36E-01
Negative	SZ	7	139	5.0% (2.2–10.5%)	—	—	SZ (Hep C-) vs. SZ (Hep C -)	1.48E-04
Positive	ZZ	2	3	66.7% (12.5–98.2%)	ZZ (Hep C+) vs. ZZ (Hep C-)	2.06E-01	ZZ (Hep C+) vs. ZZ (Hep C +)	9.07E-02
Negative	ZZ	23	82	28.0% (19.0–39.2%)	—	—	ZZ (Hep C-) vs. ZZ (Hep C -)	<2.20E-16

**Table 3:** Logistic regression for the association between genotype, hepatitis C (HCV) infection, and liver transplantation. Logistic regression was controlled for age, sex, and length of EHR data and run iteratively for hepatitis C infection status alone, genotype alone, and the interaction of genotype and hepatitis C infection.

HCV Alone			Genotype Alone		HCV + Genotype	
Odds Ratio	P-Value	Genotype	Odds Ratio	P-Value	Odds Ratio	P-Value
1.20	<2.00E-16	MM	—	—	—	—
		MS	1.004	2.81E-03	1.03	1.84E-02
		MZ	1.02	1.18E-13	1.11	6.92E-13
		SS	1.02	4.61E-02	2.21	6.05E-15
		SZ	1.04	7.81E-07	1.31	1.69E-04
		ZZ	1.31	<2.00E-16	1.23	4.70E-04

### *SERPINA1* sequencing identifies rare pathogenic variants

**Fig 2:**
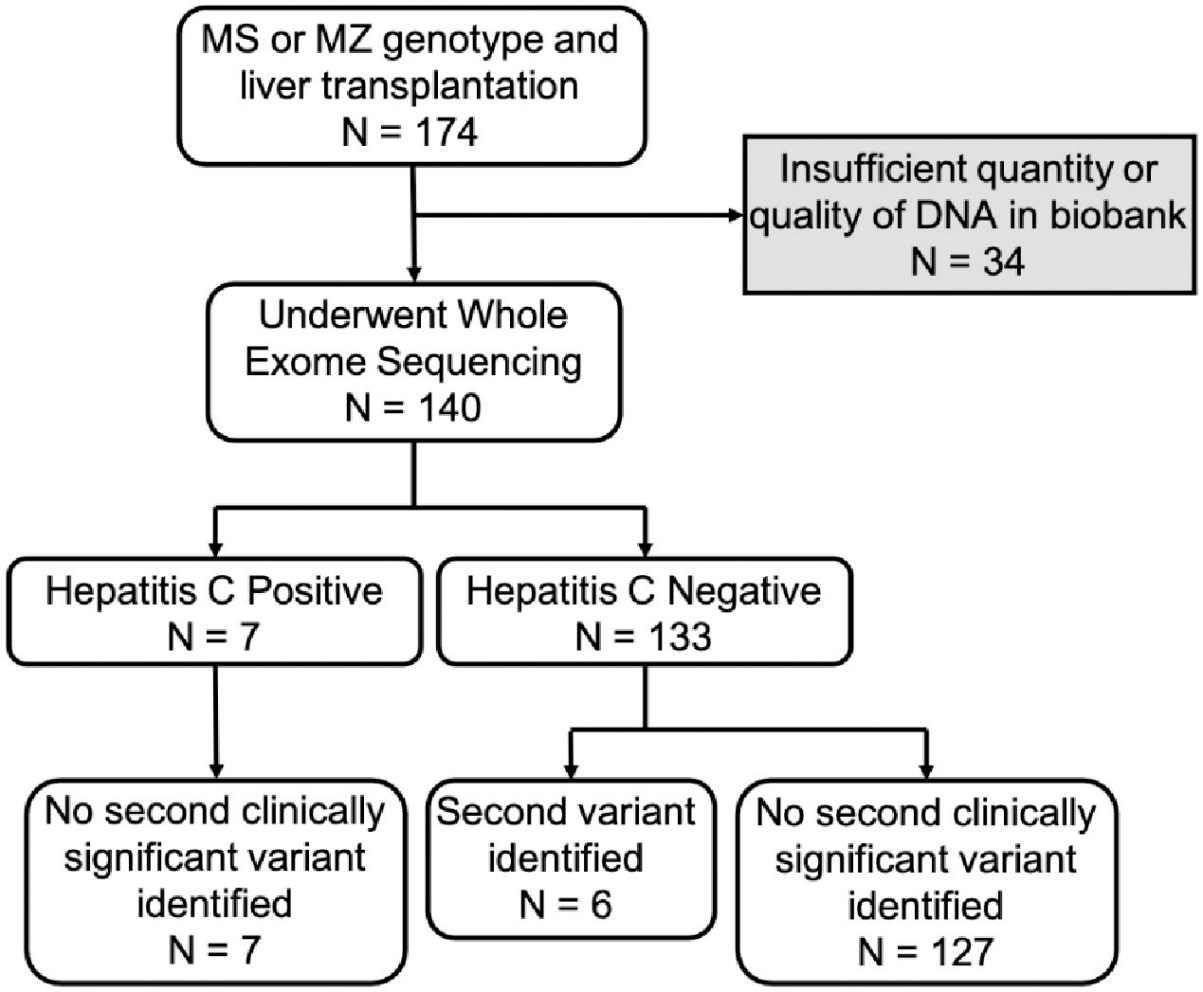
Sequencing of individuals heterozygous for a single AATD risk allele reveals six individuals without hepatitis C who have a second, rare, pathogenic variant in *SERPINA1*. Number of individuals who underwent sequencing are separated by hepatitis C infection status and sequencing results.

*SERPINA1* exons were examined in both S and Z heterozygotes to identify any rare pathogenic variants not captured through genotyping. Of the 140 individuals sequenced, seven had hepatitis C infection; none of those individuals had a second pathogenic variant identified (Fig 2). The remaining 133 individuals did not have evidence of hepatitis C infection, and *SERPINA1* sequencing revealed six individuals with a second variant of possible clinical significance (Fig 2 and Table 4). All but one of the variants identified were associated with known PI types implying risk of AATD: Z_Wrexham_, M_Wruzburg_, M_Heerlen_, M_Nichinan_, and F (individuals 2–6, Table 4. The remaining individual had a rare variant of uncertain clinical significance (individual 1, Table 4). Sequencing did not uncover a second variant of clinical significance in the other 127 individuals who lacked evidence of hepatitis C infection in their EHR (Fig 2).

**Table 4:** Genotypes, exposures, and phenotypes of individuals who had undergone liver transplant and were either found to be compound heterozygous for pathogenic variants in *SERPINA1* (individuals 1–6) or were carriers for AATD and had hepatitis C infection (individuals 7–13). Genotype data are from *SERPINA1* sequencing and the inferred PI type from the sequencing is under the genotype column, if known. Exposures include alcohol, smoking, and hepatitis C and were ascertained through chart review. Phenotypes include body mass index (BMI), chronic obstructive pulmonary disease (COPD), and alpha-1 antitrypsin (A1A) level (if measured). If the A1A level was below the reference range, the results of the tested PI typing are reported under the phenotype column.

			Genotype					Exposures			Phenotype			
Individual	Sex	Age	Nucleotide Change	Amino Acid Change	rsID	Inferred PI Type	Allele Frequency	Alcohol History	Smoking History	Hepatitis C	BMI	COPD Diagnosis	A1A Level mg/dL (Reference Range)	Tested PI Type
1	M	73	c.206C>T	p.S69F	rs199687431	—	8.37E-05	No	Former	Negative	24.2	Yes	—	—
			c.1096G>A	p.E366K	rs28929474	Z	1.26E-02							
2	F	76	c.17C>T	p.S6L	rs140814100	Z Wrexham	4.19E-05	No	Former	Negative	29.6	No	—	—
			c.1096G>A	p.E366K	rs28929474	Z	1.26E-02							
3	M	61	c.863A>T	p.E288V	rs17580	S	2.89E-02	“1 drink per week”	Former	Negative	36.6	Yes	76 (100–200)	MS
			c.1177C>T	p.P393S	rs61761869	M Wurzburg	3.00E-04							
4	M	43	c.863A>T	p.E288V	rs17580	S	2.89E-02	“3–4 drinks per week for 15 years”	Never	Negative	39.3	No	55 (83.0–199.0)	MS
			c.1178C>T	p.P393L	rs199422209	M Heerlen	1.00E-04							
5	M	60	c.514G>T	p.G172W	rs112030253	M Nichinan	6.00E-04	“Significant history”	Current	Negative	31.1	Yes	221 (100–200)	—
			c.863A>T	p.E288V	rs17580	S	2.89E-02						199 (100–200)	
6	M	62	c.863A>T	p.E288V	rs17580	S	2.89E-02	No	Never	Negative	26	No	160 (100–200)	—
			c.739C>T	p.R247C	rs28929470	F	2.10E-03						161 (83.0–199.0)	
7	F	69	c.863A>T	p.E288V	rs17580	S	2.89E-02	“4–5 beers nightly and drunk on weekends”	Current	Positive	24.6	No	“Normal”	—
8	M	56	c.863A>T	p.E288V	rs17580	S	2.89E-02	“3–4 six packs per week”	Current	Positive	27.1	No	115 (100–200)	—
9	M	61	c.1096G>A	p.E366K	rs28929474	Z	1.26E-02	“History of abuse and rehabilitation”	Current	Positive	37.9	No	—	—
10	M	59	c.863A>T	p.E288V	rs17580	S	2.89E-02	“6 pack per day”	Current	Positive	25.7	No	103 (100–200)	—
11	F	61	c.863A>T	p.E288V	rs17580	S	2.89E-02	No	Current	Positive	29.8	No	—	—
12	F	60	c.863A>T	p.E288V	rs17580	S	2.89E-02	No	Former	Positive	31.6	No	—	—
13	M	61	c.1096G>A	p.E366K	rs28929474	Z	1.26E-02	No	Never	Positive	33.8	No	94 (83.0–199.0)	—

### Chart review reveals AATD diagnosis status and supports gene-environment interaction

Manual chart review in the six compound heterozygotes for rare variants in *SERPINA1* and the seven heterozygous individuals with a history of hepatitis C infection was conducted to determine whether there was evidence of environmental risk factors for liver failure or of an AATD diagnosis missed by our pipeline (Table 4). None of the six individuals who were compound heterozygotes had an ICD code for an AATD diagnosis, but manual chart review of a clinical note for Individual 1 attributed his COPD to AATD despite the lack of A1A level, PI type, and AATD diagnosis in his EHR. Four individuals had at least one A1A level checked; two had normal levels. Two had abnormally low levels and their clinically measured PI phenotypes were both MS (Table 4).

Chart review of the seven individuals who were heterozygous for a single pathogenic allele in *SERPINA1* and had a history of hepatitis C infection revealed normal A1A levels in the two individuals in whom levels were measured (Table 4). No cases of COPD were identified despite six of them having a smoking history. Four individuals had a substantial alcohol use history, and three had BMI over 30 (Table 4). Our pipeline revealed that only one of the 140 sequenced individuals had had an ICD code for an AATD diagnosis, and sequencing did not uncover a second clinically significant variant.

### Evaluation of A1A levels as a screen for AATD

**Fig 3:**
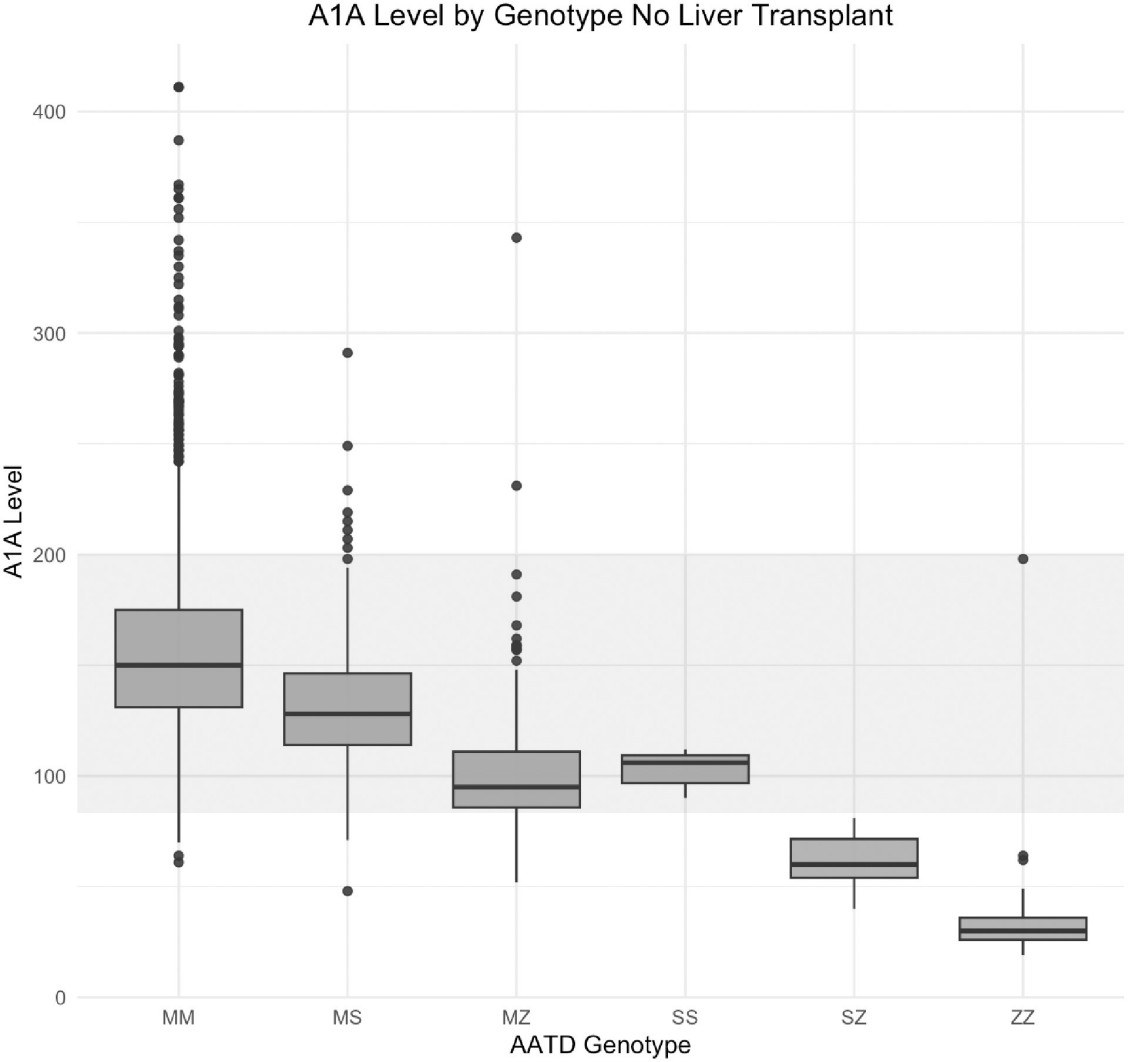
Clinically ascertained A1A levels by *SERPINA1* genotype in individuals who have not undergone liver transplantation. 2,424 individuals with genotype data also had at least one clinically obtained A1A level. Reference ranges for the tests in our database changed over time and were either 83.0–199.0mg/dL or 100.0–200.0mg/dL. The grey shading represents a reference range of 83.0-200mg/dL. The boxes represent the values between the 25^th^ and 75^th^ quantiles and the line in the box represents the median A1A value. Dots beyond the whiskers represent outliers defined as +/-1.5*inner quartile range.

We compared A1A levels to AATD risk genotypes in 2,424 individuals who have not undergone liver transplantation and for whom data were available. Generally, A1A levels decreased with increasing number and pathogenicity of *SERPINA1* variants (Fig 3). There were individuals in every genotype group with an A1A level below the reference range. However, there were also individuals in genotype groups that would be expected to have AATD with normal or elevated A1A levels (Fig 3), similar to what we found in the population of individuals with liver transplants (Individuals 5–6, Table 4).

## Discussion

In this study, genetic sequencing identified rare second pathogenic AATD alleles in six individuals with liver transplants, none of whom had a diagnostic code for AATD but one who had evidence of AATD in a clinical note. Additionally, we provide further confirmation that individuals harboring pathogenic AATD alleles, even heterozygotes, were associated with increased risk of liver transplantation. The risk of liver transplantation is further increased secondary to a gene-environment interaction between AATD alleles and hepatitis C infection. In this study, 66.7% of individuals with both hepatitis C infection and two pathogenic AATD alleles (i.e. SS, SZ, and ZZ) underwent liver transplantation compared with 8.76% of individuals with two pathogenic AATD alleles and the absence of hepatitis C infection. Similarly, 24.7% of heterozygotes for either the S or Z allele underwent liver transplantation when they had hepatitis C infection compared to 1.54% without hepatitis C infection. Other environmental exposures, such as alcohol or obesity, or other unmeasured genetic factors likely contributed to increased risk of liver transplantation for individuals who were either heterozygous or homozygous for AATD risk alleles.

*SERPINA1* sequencing facilitated identification of six individuals who are compound heterozygous for pathogenic variants. Only one of these individuals had evidence of an AATD diagnosis after full-text review of their medical records, indicating that some or all of these individuals may represent missed AATD diagnoses while acknowledging the presence of other liver disease risk factors such as obesity in these individuals likely makes their need for transplantation multifactorial. Correlation of A1A level and *SERPINA1* genotype revealed individuals with two pathogenic alleles who have normal A1A levels. There one individual with a ZZ genotype, normal A1A levels, and who was not on A1A augmentation therapy in this work (Fig 3) with a calculated sensitivity of 96.2% (25 individuals with an A1A level below threshold/26 individuals with ZZ genotype). While a study has reported as high as a 97.8% sensitivity and a 99.8% negative predictive value for the ZZ genotype with normal A1A levels [34], another study investigating diagnostic algorithms for A1A concentration found sensitivities that ranged from 61% to 95% [35]. This observation suggests that the current screening strategy in which only low levels of A1A are followed by PI typing could miss AATD diagnoses even for the most severe ZZ genotype group; this could also be the case for the individuals in this study with the M_Nichinan_S and FS PI types. The F PI type has been shown to correspond to a normal amount but dysfunctional A1A [36]. M_Nichinan_ has corresponded to decreased A1A levels [37], but this was not the case in the patient identified here. A1A is additionally an acute phase protein and can be elevated during states of acute inflammation possibly leading to a false negative AATD screen [38]. In cases with low A1A levels that reflex to PI typing, pathogenic M PI types can be missed. Two individuals had low A1A levels, and their PI typing was “MS.” However, sequence analysis revealed that they were M_Wurzburg_S and M_Heerlen_S—known pathogenic M alleles that are not easily distinguished based on size alone on the PI typing [39, 40].

End-organ damage happens in AATD secondary both to decreased protein levels that lead to insufficient neutrophil elastase inhibition in the lungs and accumulation of abnormally folded protein in the endoplasmic reticulum of hepatocytes that causes proteotoxic liver stress [41]. As both decreased A1A amount and abnormally folded protein are mechanisms of tissue damage in AATD, detection of either, even in the context of a normal A1A level, should be the goal in the diagnostic process. We have identified cases in which both the A1A level and the PI typing are fallible; consideration for the incorporation of *SERPINA1* sequencing as a part of the screening process should therefore be given to detect those alleles that may not result in decreased A1A quantity and/or an identifiable abnormality on PI typing [42]. A published AATD testing algorithm has suggested the limited inclusion of *SERPINA1* sequencing diagnostically in 2019 [43], but as the cost of sequencing has decreased and the variant interpretation process continues to improve, this should be a more viable inclusion in the AATD diagnostic process. Others have demonstrated the use of PCR-based genotyping to more readily identify pathogenic AATD variants [44], which could be an additional consideration in the screening for AATD.

Given the potential for early intervention in AATD, these data argue for increased and improved AATD screening in populations with liver disease. Individuals with even one pathogenic allele in *SERPINA1* had an increased risk for severe outcomes, especially when coupled with environmental exposures that confer risk for related tissue damage. This finding corroborates other work demonstrating the increased risk of liver disease in individuals with AATD alleles and environmental exposures (e.g. obesity, chronic alcohol use, diabetes [15, 45–47]) and highlights the need for a screening system that has improved sensitivity for detection of AATD risk genotypes that are not homozygous for the most severe Z allele. One study showed that the positive predictive value of A1A measurements was 43% for the MZ genotype group [47]. The identification of rare risk alleles beyond the more commonly assessed S and Z alleles suggests sequencing may have a role to increase diagnostic yield. Diagnosis of AATD genotype status has been shown to have a positive impact on lifestyle changes and avoidance of high-risk exposures [12, 25], but increased detection of AATD alleles is the first step in mitigating environmental risk. There is early clinical trial evidence for efficacy of an RNA interference therapeutic called Fazirsiran [48]; the presence of an efficacious therapeutic would make early detection of AATD even more important.

These data demonstrate a gene-by-environment interaction between hepatitis C and AATD risk alleles. Previous studies have investigated whether there is an enrichment of individuals with AATD risk alleles within a population of patients with liver disease and have been inconclusive; some show no association between AATD risk alleles [21–23] and liver disease and others demonstrate an enrichment of these alleles in patients with hepatitis C related liver disease [18–20, 24]. A recently published prospective study of individuals with chronic hepatitis C infection showed that there was no difference in the prevalence of Z allele heterozygotes in patients with cirrhosis compared with those without cirrhosis in two cohorts [49]. While hepatitis C and AATD risk alleles are independent risk factors for liver failure, individuals with both were at significantly increased risk of liver transplant in this study. In this study, individuals with an MZ genotype and hepatitis C infection had ~13-fold increased risk of liver transplant over those with MZ (31% vs. 2.3%) or ~3.6-fold increase over hepatitis C alone (31% vs. 19%). None of the seven individuals who were both heterozygous for either the S or Z allele and had a history of hepatitis C infection had a second pathogenic variant in *SERPINA1* identified in sequencing. While other environmental exposures like obesity and alcohol use could additionally contribute to the liver dysfunction in these patients (these risk factors have been previously implicated [15, 45–47]), the interaction between dysfunctional *SERPINA1* alleles and hepatitis C leads to the hypothesis that the mechanism by which the exposure of hepatitis C infection interacts with the underlying genetic risk of AATD is through parallel mechanisms of hepatocyte injury via endoplasmic reticulum dysfunction [41, 50]. The observed interaction between hepatitis C infection and AATD risk alleles in this study is somewhat in opposition to the finding in the recent Mücke et al. paper [49] which could be due to the differences in additional risk factors for liver dysfunction in this cohort, other population-specific differences, and/or sample size representation. While further elucidation of the clinical significance of the interaction observed in this study will be difficult to ascertain as hepatitis C is a treatable condition and fewer people will have chronic untreated hepatitis C infection, avoidance of environmental risk factors will continue to be an important part of mitigating the risks of end-organ damage related to AATD.

This study has several limitations. Use of ICD codes and phecodes can miscategorize individuals; this was the case for the individual who lacked an ICD code for AATD but had evidence of this diagnosis in manual chart review. Additionally, this study was limited to individuals of European descent. While AATD is most common in this ancestry, work should be done to investigate its applicability to other demographics. AATD is likely overrepresented and more frequently tested in this population who were seen at a quaternary-care facility that performs liver transplantation. However, the potential benefit of incorporating *SERPINA1* sequencing into routine AATD screening may be higher in general populations with liver failure. Except for the 140 individuals that underwent *SERPINA1* sequencing, we have no ability to determine the proportion of M alleles are truly functional/reference alleles and how many are dysfunctional M variant alleles. ICD codes for hepatitis C infection do not capture whether individuals were viremic and the temporal relationship between hepatitis C infection diagnosis and liver transplantation. Finally, this is a retrospective study. A randomized controlled trial comparing the current A1A measurement with reflex PI typing screening strategy with *SERPINA1* sequencing would more conclusively demonstrate superiority of sequencing over the standard measurement of A1A level with reflex PI typing for AATD screening.

In summary, increasing consideration should be given to the incorporation of *SERPINA1* sequencing into the AATD screening process to capture those individuals who may not present with a low A1A level or a detectable pathogenic allele on PI typing. This work providers further support that carriers for AATD have an increased risk of liver failure, especially in the context of other environmental risk factors (which may also include hepatitis C), and more inclusive screening measures may help mitigate AATD-related end-organ damage through lifestyle modifications and new pharmacologic interventions.

## References

[1] Teckman JH, Lindblad D. Alpha-1-antitrypsin deficiency: diagnosis, pathophysiology, and management. Curr Gastroenterol Rep. 2006;8: 14–20. doi: 10.1007/s11894-006-0059-8 16510030

[2] Perlmutter DH. Alpha-1-antitrypsin deficiency: diagnosis and treatment. Clin Liver Dis. 2004;8: 839–859, viii–ix. doi: 10.1016/j.cld.2004.06.001 15464658

[3] Teckman JH, An J-K, Blomenkamp K, Schmidt B, Perlmutter D. Mitochondrial autophagy and injury in the liver in alpha 1-antitrypsin deficiency. Am J Physiol Gastrointest Liver Physiol. 2004;286: G851–862. doi: 10.1152/ajpgi.00175.2003 14684378

[4] DeMeo DL, Silverman EK. Alpha1-antitrypsin deficiency. 2: genetic aspects of alpha(1)-antitrypsin deficiency: phenotypes and genetic modifiers of emphysema risk. Thorax. 2004;59: 259–264. doi: 10.1136/thx.2003.006502 14985567 PMC1746953

[5] Silverman EK, Miletich JP, Pierce JA, Sherman LA, Endicott SK, Broze GJ, et al. Alpha-1-antitrypsin deficiency. High prevalence in the St. Louis area determined by direct population screening. Am Rev Respir Dis. 1989;140: 961–966. doi: 10.1164/ajrccm/140.4.961 2679271

[6] Miravitlles M. Alpha1-antitrypsin deficiency: epidemiology and prevalence. Respir Med. 2000;94 Suppl C: S12–15. doi: 10.1053/rmed.2000.0852 10954249

[7] de Serres FJ, Blanco I. Prevalence of α1-antitrypsin deficiency alleles PI*S and PI*Z worldwide and effective screening for each of the five phenotypic classes PI*MS, PI*MZ, PI*SS, PI*SZ, and PI*ZZ: a comprehensive review. Ther Adv Respir Dis. 2012;6: 277–295. doi: 10.1177/1753465812457113 22933512

[8] Alpha 1-antitrypsin deficiency: memorandum from a WHO meeting. Bull World Health Organ. 1997;75: 397–415. 9447774 PMC2487011

[9] de Serres F, Blanco I. Role of alpha-1 antitrypsin in human health and disease. J Intern Med. 2014;276: 311–335. doi: 10.1111/joim.12239 24661570

[10] Janciauskiene S, Ferrarotti I, Laenger F, Jonigk D, Luisetti M. Clinical utility gene card for: α-1-antitrypsin deficiency. Eur J Hum Genet. 2011;19. doi: 10.1038/ejhg.2010.246 21248733 PMC3083625

[11] Sandhaus RA, Turino G, Brantly ML, Campos M, Cross CE, Goodman K, et al. The Diagnosis and Management of Alpha-1 Antitrypsin Deficiency in the Adult. Chronic Obstr Pulm Dis. 2016;3: 668–682. doi: 10.15326/jcopdf.3.3.2015.0182 28848891 PMC5556762

[12] Stoller JK, Brantly M. The challenge of detecting alpha-1 antitrypsin deficiency. COPD. 2013;10 Suppl 1: 26–34. doi: 10.3109/15412555.2013.763782 23527684

[13] American Thoracic Society European Respiratory Society. American Thoracic Society/European Respiratory Society statement: standards for the diagnosis and management of individuals with alpha-1 antitrypsin deficiency. Am J Respir Crit Care Med. 2003;168: 818–900. doi: 10.1164/rccm.168.7.818 14522813

[14] Teckman JH. Liver disease in alpha-1 antitrypsin deficiency: current understanding and future therapy. COPD. 2013;10 Suppl 1: 35–43. doi: 10.3109/15412555.2013.765839 23527737

[15] Carey EJ, Iyer VN, Nelson DR, Nguyen JH, Krowka MJ. Outcomes for recipients of liver transplantation for alpha-1-antitrypsin deficiency–related cirrhosis. Liver Transpl. 2013;19: 1370–1376. doi: 10.1002/lt.23744 24019185

[16] Rodriguez F, de la Roza C, Jardi R, Schaper M, Vidal R, Miravitlles M. Glutathione S-transferase P1 and lung function in patients with alpha1-antitrypsin deficiency and COPD. Chest. 2005;127: 1537–1543. doi: 10.1378/chest.127.5.1537 15888825

[17] Demeo DL, Campbell EJ, Barker AF, Brantly ML, Eden E, McElvaney NG, et al. IL10 polymorphisms are associated with airflow obstruction in severe alpha1-antitrypsin deficiency. Am J Respir Cell Mol Biol. 2008;38: 114–120. doi: 10.1165/rcmb.2007-0107OC 17690329 PMC2176135

[18] Regev A, Guaqueta C, Molina EG, Conrad A, Mishra V, Brantly ML, et al. Does the heterozygous state of alpha-1 antitrypsin deficiency have a role in chronic liver diseases? Interim results of a large case-control study. J Pediatr Gastroenterol Nutr. 2006;43 Suppl 1: S30–35. doi: 10.1097/01.mpg.0000226387.56612.1e 16819398

[19] Propst T, Propst A, Dietze O, Judmaier G, Braunsteiner H, Vogel W. High prevalence of viral infection in adults with homozygous and heterozygous alpha 1-antitrypsin deficiency and chronic liver disease. Ann Intern Med. 1992;117: 641–645. doi: 10.7326/0003-4819-117-8-641 1530195

[20] Eigenbrodt ML, McCashland TM, Dy RM, Clark J, Galati J. Heterozygous alpha 1-antitrypsin phenotypes in patients with end stage liver disease. Am J Gastroenterol. 1997;92: 602–607. 9128307

[21] Serfaty L, Chazouillères O, Poujol-Robert A, Morand-Joubert L, Dubois C, Chrétien Y, et al. Risk factors for cirrhosis in patients with chronic hepatitis C virus infection: results of a case-control study. Hepatology. 1997;26: 776–779. doi: 10.1002/hep.510260334 9303512

[22] Piekuse L, Kreile M, Zarina A, Steinberga Z, Sondore V, Keiss J, et al. Association between inherited monogenic liver disorders and chronic hepatitis C. World J Hepatol. 2014;6: 92–97. doi: 10.4254/wjh.v6.i2.92 24575168 PMC3935058

[23] Gharib AF, Karam RA, Pasha HF, Radwan MI, Elsawy WH. Polymorphisms of hemochromatosis, and alpha-1 antitrypsin genes in Egyptian HCV patients with and without hepatocellular carcinoma. Gene. 2011;489: 98–102. doi: 10.1016/j.gene.2011.08.010 21925577

[24] Settin A, El-Bendary M, Abo-Al-Kassem R, El Baz R. Molecular analysis of A1AT (S and Z) and HFE (C282Y and H63D) gene mutations in Egyptian cases with HCV liver cirrhosis. J Gastrointestin Liver Dis. 2006;15: 131–135. 16802007

[25] Aboussouan LS, Stoller JK. Detection of alpha-1 antitrypsin deficiency: a review. Respir Med. 2009;103: 335–341. doi: 10.1016/j.rmed.2008.10.006 19013782

[26] Roden DM, Pulley JM, Basford MA, Bernard GR, Clayton EW, Balser JR, et al. Development of a large-scale de-identified DNA biobank to enable personalized medicine. Clin Pharmacol Ther. 2008;84: 362–369. doi: 10.1038/clpt.2008.89 18500243 PMC3763939

[27] Denny JC, Bastarache L, Ritchie MD, Carroll RJ, Zink R, Mosley JD, et al. Systematic comparison of phenome-wide association study of electronic medical record data and genome-wide association study data. Nat Biotechnol. 2013;31: 1102–1110. doi: 10.1038/nbt.2749 24270849 PMC3969265

[28] Wu P, Gifford A, Meng X, Li X, Campbell H, Varley T, et al. Mapping ICD-10 and ICD-10-CM Codes to Phecodes: Workflow Development and Initial Evaluation. JMIR Med Inform. 2019;7: e14325. doi: 10.2196/14325 31553307 PMC6911227

[29] Wei W-Q, Bastarache LA, Carroll RJ, Marlo JE, Osterman TJ, Gamazon ER, et al. Evaluating phecodes, clinical classification software, and ICD-9-CM codes for phenome-wide association studies in the electronic health record. PLoS One. 2017;12: e0175508. doi: 10.1371/journal.pone.0175508 28686612 PMC5501393

[30] Bastarache L, Hughey JJ, Goldstein JA, Bastraache JA, Das S, Zaki NC, et al. Improving the phenotype risk score as a scalable approach to identifying patients with Mendelian disease. J Am Med Inform Assoc. 2019;26: 1437–1447. doi: 10.1093/jamia/ocz179 31609419 PMC6857501

[31] Bastarache L, Hughey JJ, Hebbring S, Marlo J, Zhao W, Ho WT, et al. Phenotype risk scores identify patients with unrecognized Mendelian disease patterns. Science. 2018;359: 1233–1239. doi: 10.1126/science.aal4043 29590070 PMC5959723

[32] Van Driest SL, McGregor TL. Pharmacogenetics in clinical pediatrics: challenges and strategies. Per Med. 2013;10. doi: 10.2217/pme.13.70 24363766 PMC3866691

[33] Alexander DH, Novembre J, Lange K. Fast model-based estimation of ancestry in unrelated individuals. Genome Res. 2009;19: 1655–1664. doi: 10.1101/gr.094052.109 19648217 PMC2752134

[34] Greulich T, Averyanov A, Borsa L, Rozborilová E, Vaicius D, Major T, et al. European screening for alpha1 -antitrypsin deficiency in subjects with lung disease. Clin Respir J. 2017;11: 90–97. doi: 10.1111/crj.12310 25919395

[35] Balderacchi AM, Barzon V, Ottaviani S, Corino A, Zorzetto M, Wencker M, et al. Comparison of different algorithms in laboratory diagnosis of alpha1-antitrypsin deficiency. Clin Chem Lab Med. 2021;59: 1384–1391. doi: 10.1515/cclm-2020-1881 33675199

[36] Sinden NJ, Koura F, Stockley RA. The significance of the F variant of alpha-1-antitrypsin and unique case report of a PiFF homozygote. BMC Pulm Med. 2014;14: 132. doi: 10.1186/1471-2466-14-132 25098359 PMC4131482

[37] Matsunaga E, Shiokawa S, Nakamura H, Maruyama T, Tsuda K, Fukumaki Y. Molecular analysis of the gene of the alpha 1-antitrypsin deficiency variant, Mnichinan. Am J Hum Genet. 1990;46: 602–612. 2309708 PMC1683626

[38] Strnad P, McElvaney NG, Lomas DA. Alpha1-Antitrypsin Deficiency. N Engl J Med. 2020;382: 1443–1455. doi: 10.1056/NEJMra1910234 32268028

[39] Silva D, Oliveira MJ, Guimarães M, Lima R, Gomes S, Seixas S. Alpha-1-antitrypsin (SERPINA1) mutation spectrum: Three novel variants and haplotype characterization of rare deficiency alleles identified in Portugal. Respir Med. 2016;116: 8–18. doi: 10.1016/j.rmed.2016.05.002 27296815

[40] Hofker MH, Nukiwa T, van Paassen HM, Nelen M, Kramps JA, Klasen EC, et al. A Pro—Leu substitution in codon 369 of the alpha-1-antitrypsin deficiency variant PI MHeerlen. Hum Genet. 1989;81: 264–268. doi: 10.1007/BF00279001 2784123

[41] Fromme M, Schneider CV, Trautwein C, Brunetti-Pierri N, Strnad P. Alpha-1 antitrypsin deficiency: A re-surfacing adult liver disorder. J Hepatol. 2022;76: 946–958. doi: 10.1016/j.jhep.2021.11.022 34848258

[42] Foil KE, Blanton MG, Sanders C, Kim J, Al Ashry HS, Kumbhare S, et al. Sequencing Alpha-1 MZ Individuals Shows Frequent Biallelic Mutations. Pulm Med. 2018;2018: 2836389. doi: 10.1155/2018/2836389 30254761 PMC6145046

[43] Franciosi AN, Carroll TP, McElvaney NG. Pitfalls and caveats in α1-antitrypsin deficiency testing: a guide for clinicians. Lancet Respir Med. 2019;7: 1059–1067. doi: 10.1016/S2213-2600(19)30141-9 31324540

[44] Schneider CV, Hamesch K, Gross A, Mandorfer M, Moeller LS, Pereira V, et al. Liver Phenotypes of European Adults Heterozygous or Homozygous for Pi*Z Variant of AAT (Pi*MZ vs Pi*ZZ genotype) and Noncarriers. Gastroenterology. 2020;159: 534–548.e11. doi: 10.1053/j.gastro.2020.04.058 32376409

[45] Veith M, Klemmer A, Anton I, El Hamss R, Rapun N, Janciauskiene S, et al. Diagnosing Alpha-1-Antitrypsin Deficiency Using A PCR/Luminescence-Based Technology. Int J Chron Obstruct Pulmon Dis. 2019;14: 2535–2542. doi: 10.2147/COPD.S224221 31819391 PMC6873957

[46] Luukkonen PK, Salomaa V, Åberg F. The Pi*MZ Allele in Alpha-1 Antitrypsin Increases Liver-Related Outcomes in a Population-Based Study. Gastroenterology. 2021;160: 1874–1875. doi: 10.1053/j.gastro.2020.10.061 33385428

[47] Schaefer B, Mandorfer M, Viveiros A, Finkenstedt A, Ferenci P, Schneeberger S, et al. Heterozygosity for the alpha-1-antitrypsin Z allele in cirrhosis is associated with more advanced disease. Liver Transpl. 2018;24: 744–751. doi: 10.1002/lt.25057 29573137 PMC6032913

[48] Strnad P, Mandorfer M, Choudhury G, Griffiths W, Trautwein C, Loomba R, et al. Fazirsiran for Liver Disease Associated with Alpha1-Antitrypsin Deficiency. N Engl J Med. 2022;387: 514–524. doi: 10.1056/NEJMoa2205416 35748699

[49] Mücke VT, Fischer J, Mücke MM, Teumer A, Koch A, Vermehren J, et al. Association of Alpha-1 Antitrypsin Pi*Z Allele Frequency and Progressive Liver Fibrosis in Two Chronic Hepatitis C Cohorts. J Clin Med. 2022;12: 253. doi: 10.3390/jcm12010253 36615054 PMC9821389

[50] Malhi H, Kaufman RJ. Endoplasmic reticulum stress in liver disease. J Hepatol. 2011;54: 795–809. doi: 10.1016/j.jhep.2010.11.005 21145844 PMC3375108

